# Pathogenesis of Zika Virus Infection

**DOI:** 10.1146/annurev-pathmechdis-031521-034739

**Published:** 2022-09-23

**Authors:** Maria I. Giraldo, Maria Gonzalez-Orozco, Ricardo Rajsbaum

**Affiliations:** 1 Department of Microbiology and Immunology, University of Texas Medical Branch, Galveston, Texas, USA; 2 Institute for Human Infections and Immunity, University of Texas Medical Branch, Galveston, Texas, USA; 3 Current affiliation: Center for Virus-Host-Innate-Immunity; Rutgers Biomedical and Health Sciences, Institute for Infectious and Inflammatory Diseases; and Department of Medicine, New Jersey Medical School, Rutgers, The State University of New Jersey, Newark, New Jersey, USA

**Keywords:** Zika virus, tropism, ubiquitin, antagonism of immune responses, pathogenesis, TRIM7

## Abstract

Zika virus (ZIKV) is an emerging virus from the *Flaviviridae* family that is transmitted to humans by mosquito vectors and represents an important health problem. Infections in pregnant women are of major concern because of potential devastating consequences during pregnancy and have been associated with microcephaly in newborns. ZIKV has a unique ability to use the host machinery to promote viral replication in a tissue-specific manner, resulting in characteristic pathological disorders. Recent studies have proposed that the host ubiquitin system acts as a major determinant of ZIKV tropism by providing the virus with an enhanced ability to enter new cells. In addition, ZIKV has developed mechanisms to evade the host immune response, thereby allowing the establishment of viral persistence and enhancing viral pathogenesis. We discuss recent reports on the mechanisms used by ZIKV to replicate efficiently, and we highlight potential new areas of research for the development of therapeutic approaches.

## 1. INTRODUCTION

Zika virus (ZIKV) is an enveloped, positive-strand, single-stranded, nonsegmented RNAvirus that belongs to the *Flaviviridae* family. The virus genome is composed of a single open reading frame that encodes a single polyprotein, which is processed by cellular enzymes and viral proteases into 10 proteins. These viral proteins include three structural proteins [envelope (E), membrane (M), and capsid (C)] that form the viral particle and seven nonstructural (NS) proteins (NS1, NS2A, NS2B, NS3, NS4A, NS4B, and NS5), which are necessary for processing, replication, and assembly of new viruses (1). ZIKV can be transmitted by mosquitoes (*Stegomyia aegyptus and Stegomyia albopictus*) (2) and human sexual contact (3) as well as vertically from infected pregnant women to their embryos (4).

In 1947, ZIKV was isolated in Uganda from a sentinel rhesus monkey (5). A few sporadic human cases were reported in Africa and Asia between 1964 and 2007 (6–9) without major health consequences. In 2007, the first outbreak of ZIKV causing disease was reported in Micronesia (10), and in 2013 an outbreak was reported in French Polynesia that later spread to several Pacific islands (9). In 2015, ZIKV infections were reported in Brazil, and this outbreak sparked major concerns due to the increased association with microcephaly cases in newborns from infected mothers (11). This outbreak spread to most countries in south and central America. ZIKV continues to circulate in the tropical and subtropical regions of approximately 87 countries and territories where there has been evidence of endemic mosquito-borne transmission of ZIKV during recent years (12–14). Over the past few decades, the number of people infected with ZIKV has risen steadily due to the expansion of urban populations, global travel and commerce, climatic change, and a paucity of mosquito control programs. The coronavirus disease 2019 (COVID-19) pandemic has also likely resulted in underreporting due to lockdowns and COVID-19 patients overwhelming the health-care system.

Pregnant women are vulnerable to viral infections during the first and second trimesters when there is an elevated risk for congenital damage to the fetus. Infections of ZIKV in pregnant woman can lead to spontaneous abortion, intrauterine growth restriction, and microcephaly in newborns (15, 16), which results from the ability of ZIKV to infect placental cells and neural precursors in the fetus (15, 16). Approximately 5–15% of babies born to infected women during pregnancy have reported birth defects, and there is evidence of ZIKV-related complications that can lead to what is known as congenital Zika syndrome (CZS). These congenital malformations, which include cortical atrophy with microcephaly and functional impairments such as dysphagia and epilepsy, can occur after symptomatic and asymptomatic infection (17–19). A recent study followed a large cohort of live-born children from ZIKV-infected mothers in Brazil during the last outbreak and found that these children were at least 10 times more likely to die than children without CZS (19). Although attention to ZIKV infection has waned recently, ZIKV cases are likely underreported, and there is the risk of reemergence once current travel restrictions are lifted. There is no antiviral therapy for controlling the disease, and only a few vaccines are in early clinical development. In this review, we discuss recent discoveries on the molecular mechanisms used by ZIKV to escape the immune response as well as ZIKV tropism, persistence, and mechanisms leading to pathogenesis.

## 2. CHARACTERISTICS OF THE IMMUNE RESPONSE TO ZIKV

ZIKV has the ability to evade the innate and adaptive immune response to efficiently infect different cells and tissues causing pathology. Although significant efforts have been made to identify host factors that are involved in the pathogenesis of ZIKV, much is still unclear. Several reports have shown that ZIKV can activate the host immune response, leading to protection (20); however, inflammation can also have detrimental effects on the embryo during pregnancy (21).

Therefore, there are multiple mechanisms of pathogenesis, which may involve ZIKV either dampening an effective antiviral response leading to increased virus replication or indirectly inducing immunopathology due to dysregulated immune responses. There is vast literature on the role of specific ZIKV proteins in the antagonism of the innate immune response at different steps of the viral replication cycle, and this has been summarized in excellent recent reviews (21–25). In this section, we focus our discussion on novel aspects of ZIKV immune antagonism and the experimental evidence associating these effects with potential pathology in the embryo.

### 2.1. Manipulation of Intrinsic Defenses by ZIKV

The early antiviral response relies on innate responses as well as intrinsic immunity to defend cells against infection. Intrinsic defenses allow cells to respond more quickly to infection than innate responses, because they do not require de novo protein synthesis. Instead, intrinsic defenses are molecules that are already present in levels high enough to act directly and almost immediately upon infection or that can be rapidly activated upon a signal, helping to interfere with the viral replication cycle. These mechanisms include autophagy, apoptosis, and RNA interference (RNAi). However, as is the case for other immune responses, ZIKV can also manipulate these host intrinsic defenses to enhance replication and pathogenesis.

#### 2.1.1. Autophagy and ZIKV.

**Figure 1:**
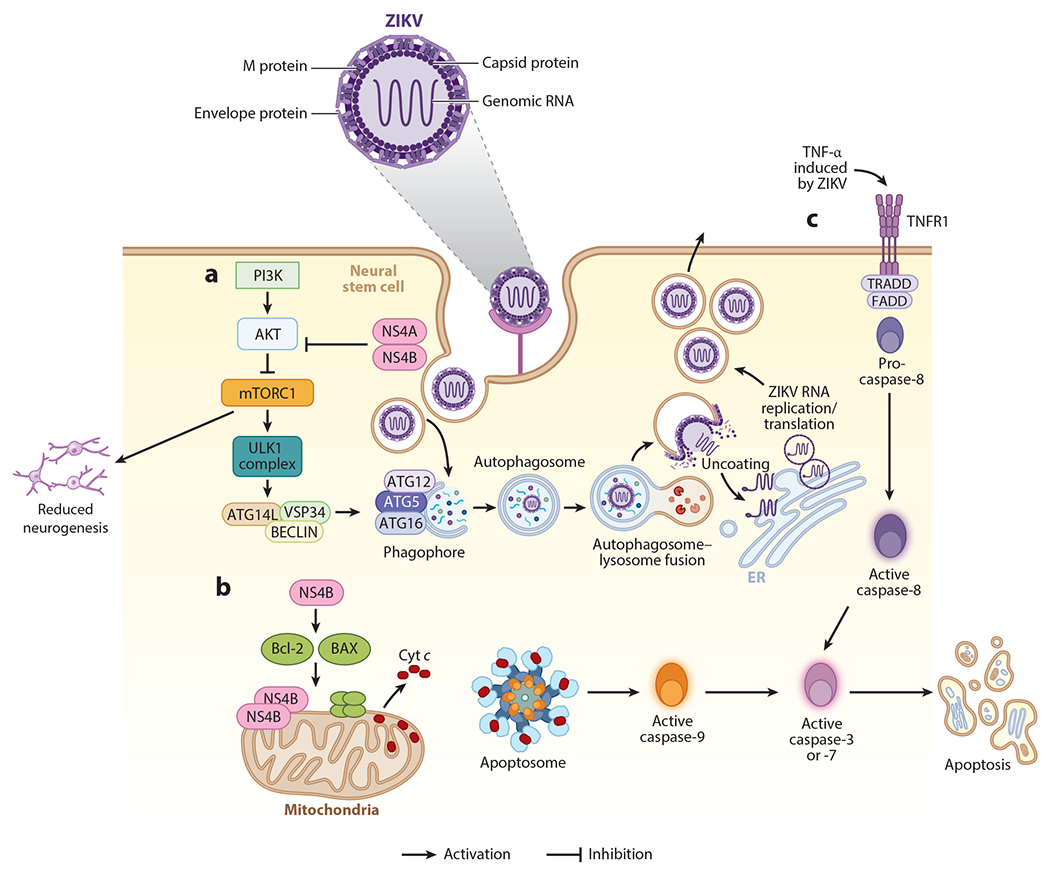
Intrinsic immunity and mechanisms of antagonism by Zika virus (ZIKV). (*a*) ZIKV proteins NS4A and NS4B can block the activation of AKT and subsequently lead to the inactivation of mTORC1, allowing activation of the ULK complex by AMP-activated protein kinase and the initiation of the autophagosome formation through ATG16. ZIKV is internalized via endosomes and autophagosomes. The autophagosome fuses with the lysosome, leading to the acidification of the autophagolysosome and activation of proteases to degrade the viral components. This process could be beneficial for ZIKV because acidification allows fusion of the ZIKV envelope with the endosomal membrane, allowing the viral RNA to reach the cytoplasm. The inhibition of the AKT-mTOR pathway has an adverse effect in the proliferation of the infected cells, with catastrophic consequences in the formation of the neuronal network and tissue development. (*b*) ZIKV NS4B protein can localize near the mitochondria membrane and activate the Bcl-1-associated X protein (BAX) to form a complex with Bcl-2,which interacts with the mitochondrial voltage-dependent anion channel, leading to loss in membrane potential and release of cytochrome c (Cyt *c*). This in turn leads to the activation of the intrinsic apoptosis pathway, with the activation of caspase-9 and caspase-3. (*c*) The activation of the extrinsic pathway can be a bystander effect, since it is mediated by the activation of cell death receptors FAS and TNFR1, and it is induced by external signals such as tumor necrosis factor alpha (TNF-α), leading to the activation of caspase-8 and caspase-3 to induce cell apoptosis.

Recent evidence suggests that autophagy may play important roles both in promoting virus replication and in host defense against ZIKV (26). Autophagy is a cellular mechanism that occurs in mammalian cells and has many effects on immunity and inflammatory processes. Autophagy is a highly complex pathway playing a significant role in the digestion and degradation of intracellular pathogens via lysosomes, including viruses, promoting antiviral responses (26–29). One autophagy pathway involves the inhibition of the AKT kinase and the mTORC1 complex. Inhibition of AKT-mTORC1 allows recruitment of autophagic factors to form an autophagosome/lysosome that can degrade engulfed viruses. Although autophagy can promote clearance of intracellular infections, flaviviruses have developed mechanisms to manipulate autophagy pathways to amplify their replication cycles (26, 30, 31). ZIKV NS4A and NS4B proteins induce autophagy by suppressing the host AKT-mTOR signaling pathway in human fetal neural stem cells (fNSCs), by inhibition of PI3K activity and possibly by modulating posttranslational modifications of AKT (32). Since the PI3K-AKT pathway is also important in brain development (33), inhibition by ZIKV can result in defective neurogenesis (32) (Figure 1).

ZIKV-induced autophagy is not restricted to fNSCs and has also been demonstrated in other relevant cell types, including human skin fibroblasts, human trophoblasts, human umbilical vein endothelial cells, and human cytotrophoblasts (30, 34, 35). The evidence linking autophagy with proviral effects derives from experiments in which treatment with drugs that activate autophagy also increased ZIKV replication, while inhibitors of autophagy reduced viral replication, and this was demonstrated in different cell types (30, 34, 35). Most importantly, ZIKV infection of *Atg16l1* (an essential autophagy gene) knockout mice resulted in reduced viral transmission to the embryos of pregnant mice; infection of maternal organs was not affected, indicating that autophagy plays a cell-type-specific proviral role in physiological conditions (34). Together, these studies indicate that, at least in vivo, the proviral roles of autophagy are dominant over its antiviral roles. Indeed, inhibitors of autophagy have been proposed as a potential viable option to control ZIKV infection in pregnant women (34).

#### 2.1.2. Apoptosis and ZIKV.

Like autophagy, apoptosis is another cellular process that continuously regulates cellular homeostasis. Apoptosis is a well-established antiviral mechanism that helps reduce virus replication and dissemination to adjacent cells. Some strains of ZIKV have the ability of inducing apoptosis to increase replication in SY5Y neuroblastoma cell lines (36). This induction activates the classic mitochondrial apoptotic pathway with the recruitment of the proapoptotic protein Bax to the mitochondria. It is possible that during ZIKV infection, NS4B protein accumulates in the outer mitochondrial membrane to permeabilize and release proapoptotic factors, such as cytochrome c, to then activate caspase-3 and caspase-9, ultimately leading to cell death (36). ZIKV infection of human neural progenitor cells can lead to cell death via apoptosis through the activation of caspase-3, -7, -8, and -9 (37–39) (Figure 1).

#### 2.1.3. RNA interference and ZIKV.

RNAi is an ancient intrinsic posttranscriptional gene silencing process and host defense mechanism that has been extensively studied and demonstrated in invertebrates (40). It has also been proposed to act as an innate antiviral response in mammals (41), although this has been controversial and debated (42). During viral infection, RNA replication intermediates are cleaved by the host endoribonuclease Dicer into short interfering RNAs that bind Argonaute proteins for virus silencing (40) and can also produce virus-derived small interfering RNAs (vsiRNAs) (43). The debate over whether mammalian cells can produce antiviral RNAi was recently clarified with the discovery of an alternative spliced form of Dicer called antiviral Dicer (aviD). aviD is highly expressed in murine and human stem cells and inhibits ZIKV replication by an RNAi-dependent mechanism (44). This evidence sheds some light on previous observations of ZIKV manipulation of the RNAi machinery. For example, the capsid protein of ZIKV was shown to inhibit Dicer activity, and a recombinant ZIKV capsid mutant ZIKV-H41R lost its ability to antagonize Dicer. Infections with this mutant virus resulted in increased production of large amounts of vsiRNAs and appeared less pathogenic (45). The production of vsiRNAs by ZIKV was dependent on RNAi machinery proteins (46, 47), and enoxacin, a known RNAi enhancer, showed strong antiviral activity and reduced phenotypes associated with microcephaly (46). Although ZIKV infection revealed the presence of 29 vsiRNAs in the ZIKV genome, with vsiRNA-p18 being the most abundant in neural stem cells (47), the physiological antiviral role of these vsiRNAs is still unclear.

### 2.2. Innate Immunity

**Figure 2:**
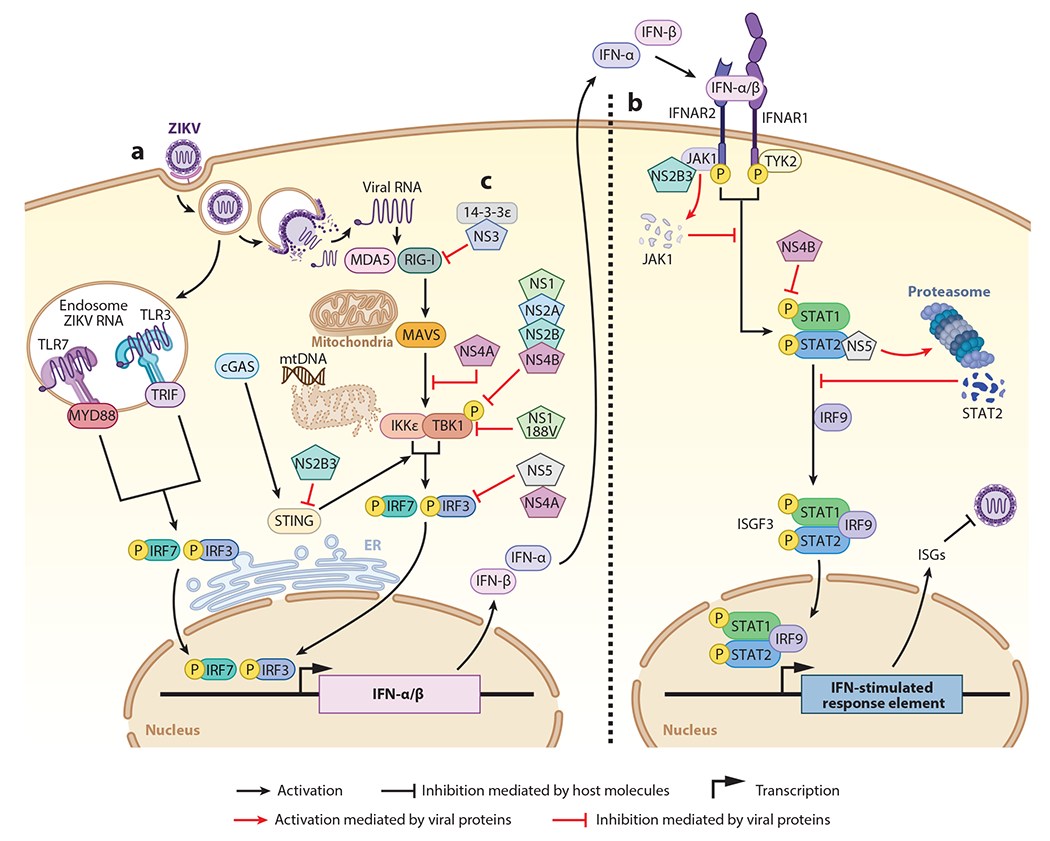
Innate immune activation and evasion mediated by Zika virus (ZIKV). (*a*) After infecting a cell, ZIKV RNA is recognized by multiple cell pattern recognition receptors, such as endosomal Toll-like receptors (TLRs) and cytosolic RIG-I and MDA5. Viral recognition triggers downstream signaling pathways that culminate in the activation and translocation to the nucleus of transcription factors IRF3 [interferon (IFN)-regulatory factor 3] and IRF7 to induce the transcription of type-I IFNs (IFN-α and IFN-β). (*b*) The production of IFNs is important to reduce the viral spread to surrounding cells, as they can signal in an autocrine or paracrine manner through the IFN receptor (IFNAR1 and IFNAR2). IFN signaling involves the JAK1 and TYK2 kinases that phosphorylate STAT1 and STAT2, which then together with IRF9 form the ISGF3 complex. ISGF3 translocates to the nucleus to promote transcription of IFN-stimulated genes (ISGs), which many have antiviral activity. (*c*) ZIKV antagonizes the antiviral response. The viral protein NS3 binds to 14-3-3ε and prevents the translocation of RIG-I and MDA5 to the mitochondria for interaction with MAVS. NS4A binds to MAVS, preventing the association with RIG-I and MDA5. NS1, NS2A, NS2B, and NS4B interact with TBK1, reducing its phosphorylation, and inhibit the IFN-I production pathway. NS2B3 targets STING and blocks the signaling through TBK1. NS5 and NS2A act later in the pathway, inhibiting IRF3 and preventing its translocation to the nucleus and the induction of IFN-I. In the IFN-I signaling pathway, NS2B3 binds to JAK1 and induces its degradation, preventing the activation of STAT proteins. NS4B inhibits the pathway by inhibiting the phosphorylation of STAT1, while NS5 binds to STAT2 and promotes its proteasomal degradation. All of these mechanisms prevent induction of ISGs and diminish the antiviral response.

The innate immune system has the ability to detect ZIKV infection via host pattern recognition receptors (PRRs). Viral recognition by PRRs triggers a complex web of signaling pathways that culminate in the expression of type I interferons (IFN-I) and other proinflammatory cytokines. IFN-I then signals in an autocrine or paracrine manner to induce a large number of IFN-stimulated genes (ISGs), which are the final effectors of the antiviral response (25, 48). The most studied classes of PRRs are the RIG-I-like receptors (RLRs), which include RIG-I, MDA5, and LGP2, and the Toll-like receptor (TLR) families. These PRRs can recognize pathogen-associated molecular patterns such as viral RNA from ZIKV (49, 50). TLRs are sensors expressed by epithelial cells and immune cells, and they have the function of inducing IFN production in the presence of a wide variety of viruses, allowing viral restriction (51). For example, plasmacytoid dendritic cells (DCs) express high levels of TLR7, which recognizes single-stranded RNA (ssRNA), or TLR9, which recognizes unmethylated CpG sequences in DNA, and activate signaling through the myeloid differentiation factor 88 pathway. This, in turn, activates the interleukin (IL)-1 receptor-associated kinase family of kinases, ultimately leading to phosphorylation of IFN-regulatory factor 3 (IRF3) and IRF7, which are required for IFN-I production (52). TLR3, which is expressed in multiple cell types including myeloid DCs and epithelial cells, recognizes double-stranded RNA (dsRNA) produced during viral infection. TLR3 triggers the pathway via the TIR domain–containing adapter-inducing IFN-β (TRIF) protein, which uses its TIR domain to recruit tumor necrosis factor (TNF) receptor–associated factors and subsequently activate IRF3/7 to promote IFN-I production (53) (Figure 2).

While recognition of viral RNA by TLRs occurs in the endosomes, when released to the cytoplasm, viral RNA can be recognized by the RLR pathway. Binding of viral RNA to RIG-I triggers conformational modifications on RIG-I to expose its CARD domain to allow interaction with the mitochondrial adaptor protein MAVS (54). In turn, multiple signaling factors are activated downstream of MAVS, including the kinases TBK1 and IKKε, which phosphorylate transcription factors IRF3 and IRF7 for induction of IFN-I transcripts (55).

After IFN-I is induced by either the RLR or the TLR pathway, IFN-I binds to its receptor (IFNAR1 and IFNAR2) to transduce the signal through Janus kinases (JAK1 and TYK2) that phosphorylate transcription factors STAT1 and STAT2, which together with IRF9 form the ISGF3 complex, to ultimately induce ISGs (52) (Figure 2).

Another important pathway in IFN production is the cyclic guanosine monophosphate (GMP)–adenosine monophosphate (AMP) synthase–stimulator of IFN genes (cGAS-STING) pathway, which is mainly characterized by recognizing cytoplasmic DNA (either its own or foreign) (56). cGAS initiates the synthesis of cyclic GMP-AMP, which in turn binds to the endoplasmic reticulum adaptor protein STING, which recruits TBK1 for subsequent activation of IRF3 leading to the production of IFN-I (56). Dengue virus (DENV) and ZIKV have been shown to trigger the cGAS pathway by a mechanism that involves mitochondrial damage and release of DNA that can be detected by cGAS (56–58). Zika virus is sensitive to activation of these pathways, and this is especially evidenced by the fact that ZIKV does not replicate well in immunocompetent mice but does replicate well in IFN receptor knockout mice (A129) (59). Therefore, to establish productive infections, multiple ZIKV proteins have been shown to block different steps of the interferon pathway (25, 60) (Figure 2).

#### 2.2.1. ZIKV and Toll-like receptors.

The expression of different PRRs varies between cell types and is key to the production of the pro- or anti-inflammatory response. ZIKV has the ability to induce IFN-I production by activating TLR3, TLR7, RIG-I, and MDA5. In the case of TLRs and ZIKV, the ssRNA of the virus can potentially be recognized by TLR7. On the other hand, it can activate TLR3 through the dsRNA intermediate that is generated during genome replication. A study demonstrated that DCs present in the skin express high levels of TLR3 and TLR7 and play an important role in the immune response against ZIKV infection (35). ZIKV infection considerably increased the expression of TLR3 and therefore the production of IFN-α and IFN-β in infected cells. A significant increase in viral replication could also be evidenced by using the small interfering RNA (siRNA) of TLR3, but no effect on the expression of IFN-I mRNA was detected (35). Also, TLR3 was previously associated with recognition of ZIKV in an organoid and neurosphere model derived from human embryonic stem cells of fetal brain development, where activation of apoptosis and decreased neurogenesis was demonstrated (61). It has also been shown that using the TLR7/8 receptor agonist R848 (resiquimod) in monocyte-derived macrophages limits ZIKV replication by increasing the expression of several ISGs (62) (Figure 2).

#### 2.2.2. ZIKV and the cytoplasmic sensors RIG-I, MDA5, and cGAS.

RIG-I and MDA5, which have been extensively studied for their role as intracellular sensors for ZIKV, have the ability to recognize structured ssRNA or dsRNA of viral origin generated during replication in the cytoplasm (55). In human DCs, ZIKV infection induces transcription of RIG-I and MDA5. This is a result of a robust IFN-I response because both RIG-I andMDA5 are themselves induced by IFN and, thus, are ISGs. ZIKV replication in human DCs was strongly limited when cells were treated with the hepatitis C virus RNA-derived RIG-I agonist (63), indicating that ZIKV is sensitive to the antiviral activity of IFN-I, although reduced secretion of inflammatory cytokines was observed during ZIKV infection (63). This is further supported by the requirement for IFN-I receptor knockout mice when studying in vivo infection with ZIKV (59). In human trophoblasts, RIG-I and MDA5 inhibit ZIKV replication via IFN-I induction and, as expected, were completely dependent on the presence of MAVS (64). In A549 cells, RIG-I signaling is responsible for generating the immune response in the early stages of ZIKV infection, while stimulation of MDA5 occurs during late stages (65). Additionally, ZIKV RNA can be detected by RIG-I and MDA5 sensors to induce a protective response against ZIKV in a cell-type-dependent manner (66), and this recognition may need the concerted function of other helicases, such as the recently reported DHX16 (67). ZIKV can also indirectly activate the DNA sensor cGAS via DNA released from mitochondria damaged during virus infection to induce IFN-I production (58) (Figure 2).

#### 2.2.3. Evasion of the innate immune response.

Viruses generally use their proteins as the main evasion mechanism of the immune response to ensure viral replication. Viral proteins can target important factors to interfere with both IFN production as well as signaling pathways. For example, one of the most extensively studied mechanisms is the inhibition of the JAK-STAT signaling pathway by NS5. ZIKV NS5 binds to the coiled-coil domain of STAT2 (68), promoting proteasomal degradation and inhibition of ISG induction (69, 70). It is important to mention that this effect is species specific because NS5 degrades humans but not mouse STAT2 (69, 71), and an immunocompetent transgenic knock-in mouse expressing human STAT2 instead of mouse STAT2 is susceptible to ZIKV replication and pathogenesis (72). Interestingly, this species-specific degradation of STAT2 by NS5 is conserved between different flaviviruses including yellow fever virus (YFV), DENV and ZIKV; however, it appears that only YFV-NS5 requires IFN-I signaling to bind and degrade STAT2 (73, 74). For ZIKV-NS5, the cryogenic electron microscopy–determined structure of STAT2 complexed with NS5 revealed two potential different mechanisms of inhibition, one in which NS5 methyltransferase and RNA polymerase domains interfere with STAT2 binding to NS5 and a second in which interaction with the N-terminal domain of STAT2 might be involved in the subsequent proteasomal degradation (75). ZIKV NS5 has also been found to interact with IRF3, which can also result in reduced IFN-β induction (60).

Other viral proteins are also known to antagonize multiple different parts of IFN pathways. ZIKV NS4A binds MAVS and prevents its association with RIG-I and MDA5 (64). Several studies have also shown that ZIKV proteins such as NS1, NS2A, NS2B, and NS4B interact and inhibit TBK1 function, reducing the phosphorylation of transcription factors and IFN induction (60, 70). Importantly, some effects have been found to be specific of more pathogenic strains of ZIKV. For example, the inhibition of IFN-I by NS1 was restricted to a single mutation on NS1 that appeared in strains with higher epidemic potential, including a strain isolated in Puerto Rico (PRVABC-59) during the ZIKV epidemic in 2015 (60). This suggests that ZIKV can acquire novel mutations during epidemics that enhance its pathogenicity and transmission by increasing the ability to antagonize innate antiviral pathways. Although the mechanism by which NS1 antagonizes IFN-I was narrowed down to targeting of TBK1, the detailed molecular mechanism was not elucidated. Since NS1 can also interact with NS4B, and this interaction was shown to be affected by ubiquitination of NS1 for DENV (76), it will be interesting to see whether NS1 ubiquitination is involved in promoting interactions with TBK1. In addition to TBK1, ZIKV NS1 can activate inflammasomes to avoid the caspase-1 degradation by the proteasome. In this context, NS1 facilitates the cleavage of cGAS by caspase-1, which blocks the cGAS-STING pathway and IFN induction (58). STING-dependent signaling can also be targeted by the ZIKV NS2B3 protease (77). In human cells and some cells of primate species infected with ZIKV, the NS2B3 proteases of the virus have the ability to cleave STING, which leads to decreased production of IFN-I and subsequently increased viral replication. This has also been observed in other flaviviruses such as West Nile virus (WNV), Japanese encephalitis virus, and DENV, but not YFV (77–79). These findings suggest that ZIKV uses this evasion mechanism and that it is important in tissue and species tropism. The ZIKV NS2B3 complex can also inhibit IFN-I signaling by binding and degrading JAK1, preventing STAT phosphorylation (70) (Figure 2).

Studies on ZIKV NS4B have shown additional roles for the protein in IFN antagonism, including that it can inhibit IFN signaling and IFN-γ-activated site transcription. The mRNA expression levels of the *Isg15* and *Oas1* genes were reduced in the presence of NS4B as a consequence of suppressed phosphorylation of STAT1 and reduced nuclear transport of STAT1 and STAT2 (80).

Several reports indicate that the two close relatives of ZIKV, DENV and WNV, use a conserved mechanism for immune evasion (81, 82). One report demonstrated that the ZIKV NS3 protein binds to the RLR trafficking protein of the family 14-3-3, preventing the translocation of RIG-I and MDA5 to the mitochondria for the activation of MAVS. This could also be confirmed using mutant viruses deficient in the NS3 protein, which generated an increase in the antiviral response (82).

Another unconventional mechanism of IFN antagonism includes inhibition of IFN-I induction via ZIKV subgenomic flavivirus RNA (sfRNA), which appears to take place by specifically targeting RIG-I but not MDA5 (83). The extent by which the sfRNA may act as an IFN-I antagonist may depend on the specific viral strains, as it has been observed for some DENV strains with increased fitness (84). Overall, these different mechanisms of innate immune antagonism are examples of how ZIKV has adapted to specific host environments to establish efficient infections, and mutations appearing in new strains could provide ZIKV with enhanced epidemic potential as well as altered cellular tropism and pathogenesis.

### 2.3. Adaptive Immune Response

In general, the adaptive immune response to viral infections includes the activation of CD8 cytotoxic T cells that kill the infected cells and CD4 T helper cells that develop toward a Th1 phenotype producing effector cytokines such as IFN-γ and TNF-α. B cells are also a central player in the antiviral response, because they produce neutralizing antibodies (Nabs) that prevent the interaction of the virus with host cells, thus preventing infection. Nabs also mediate complement activation and antibody-dependent cellular cytotoxicity, leading to lysis of infected cells (23). As discussed above, mouse models of ZIKV require suppression of the IFN response to establish ZIKV infection. However, immunocompetent mouse models have also been used to unveil the components of the adaptive immune response present during ZIKV infection. These studies have shown that ZIKV infection induces T cell activation with proliferation of CD4^+^ and CD8^+^ T cells seven days post infection, and T cell depletion had very minor effects. To reveal the role of the adaptive response, however, it was still necessary to block the IFN pathway. Blocking the IFN receptor in the absence of an adaptive immune response using *Rag1*^−/−^ mice, which lack both T cells and B cells, increased weight loss and virus titers, indicating that in mice the adaptive response is critical only when IFN-I is suppressed (85). Another study demonstrated that ZIKV-infected wild-type (WT) immunocompetent C57BL/6 mice exhibit a self-limited infection that resulted in mild symptoms. Activation of the adaptive immune response induced proliferation of CD4 T cells expressing the transcription factor T-bet, leading to a Th1 response with increased production of cytokines such as IFN-γ, IL-2, and TNF-α. CD8 T cells were also activated to produce IFN-γ, TNF-α, and granzyme B (86, 87). These studies show that, although very limited virus replication is observed in immunocompetent mice, ZIKV can induce cellular adaptive immune responses during the infection.

In the past decade, researchers have joined efforts to understand cross-reactive T cell–mediated protection against ZIKV infection. To determine the characteristics of this protection, they have examined the cross protection of T cells in close relatives of the flavivirus family, specifically DENV. Certain protective responses identified during ZIKV infection could be mediated by CD8 T cells that specifically recognize structural proteins such as E, precursor membrane (prM), and C in non-DENV-exposed individuals (88), while CD4 T cell responses are primarily directed against nonstructural proteins such as NS3, NS4B, and NS5 after exposure to DENV (89, 90). Both activations ultimately generated IFN-γ responses. However, another study showed that some CD4 T cells could also recognize ZIKV C and E proteins without previous exposure to DENV (91).

#### 2.3.1. The humoral response.

The humoral response is also critical for functional immunity against ZIKV. Several studies have shown that neutralizing antibodies specific for the envelope domain EDIII provide protection against lethal ZIKV infection, although some antibodies that cross-react with DENV can enhance disease (92–94). Thus, these reports provide insight into the role of memory B cells in modulating the response, which would allow the design of vaccines directed to the envelope protein. However, careful attention should be paid to the potential disease enhancement caused by some cross-reacting antibodies.

#### 2.3.2. Pathogenic adaptive immune response during ZIKV infection.

While a regulated adaptive immune response can protect against infection, an exacerbated response can contribute to tissue damage and pathology. Infections with YFV and WNV, close relatives of ZIKV, promote infiltration of CD8 T cells, which correlate with the tissue damage and neurological symptoms in mouse models, despite their critical role in controlling viral load (95). Similar results have been obtained for ZIKV For example, infection of neonatal WT mice with ZIKV leads to increased recruitment and activation of CD8 T cells and the production of IFN-γ, granzyme B, and perforin. The levels of these inflammatory mediators correlated with the neurodegeneration observed predominantly in the cerebellum (96). Another study using IFNAR knockout mice demonstrated the importance of CD8 T cells in the immunopathogenesis of ZIKV. Depletion of CD8 T cells resulted in increased survival and significantly decreased paralysis despite increased viral load in the brain. In contrast, depletion of CD4 T cells resulted in complete paralysis (97). These results suggest that CD8 T cells play a pathogenic role during infection in the CNS, while CD4 T cells appear to have a regulatory role (97). Further studies are needed to understand the mechanisms of adaptive immune activation during virus infection leading to enhanced pathogenesis in target tissues.

## 3. MECHANISMS OF ZIKV PERSISTENCE, TISSUE TROPISM, AND PATHOGENESIS

One of the most intriguing aspects of ZIKV pathogenesis is its unique characteristic of causing defects in embryos and, more specifically, its association with microcephaly. One likely explanation is the ability of ZIKV to infect neural progenitor cells and cause cellular apoptosis (36–39, 61, 98, 99). In addition, the fact that ZIKV infects placenta cells and cells of reproductive tissues very efficiently further increases the ability of ZIKV to reach and disseminate in the embryo to ultimately cause damage. While it is clear that ZIKV has a preference to infect neural cells in the developing embryo, it is less clear what are the molecular mechanisms that provide ZIKV the unique preference for these tissues. One possibility could be the tissue-specific expression of cellular receptors needed for ZIKV internalization, but so far, no major or single specific receptor has been clearly demonstrated as required for ZIKV entry, at least in physiological conditions. A few candidate receptors have been proposed in cell culture studies, including AXL, DC-SIGN, Tyro3, and TIM-1 (35). AXL is probably one of the most extensively studied in cells and in vitro and has been proposed as a strong candidate receptor for ZIKV (100); however, its role in vivo using *Axl*^−/−^ mice has mostly cast doubt on its role as the main receptor (98, 100). ZIKV replication was detected in testis and epidermis of *Axl*^−/−^ mice (101) as well as in the brains and spleens of *Axl*^−/−^ pregnant mice or brain and placenta tissue of the embryos (102), at comparable levels with WT mice. It is possible that multiple receptors play a role in ZIKV entry or that multiple internalization pathways may play redundant roles, which could obscure interpretation of the results in these studies. Regardless of which receptor is dominant for ZIKV entry, it is also possible that other host factors play essential roles in determining ZIKV tropism. These other host factors may also contribute to persistence of ZIKV.

**Figure 3:**
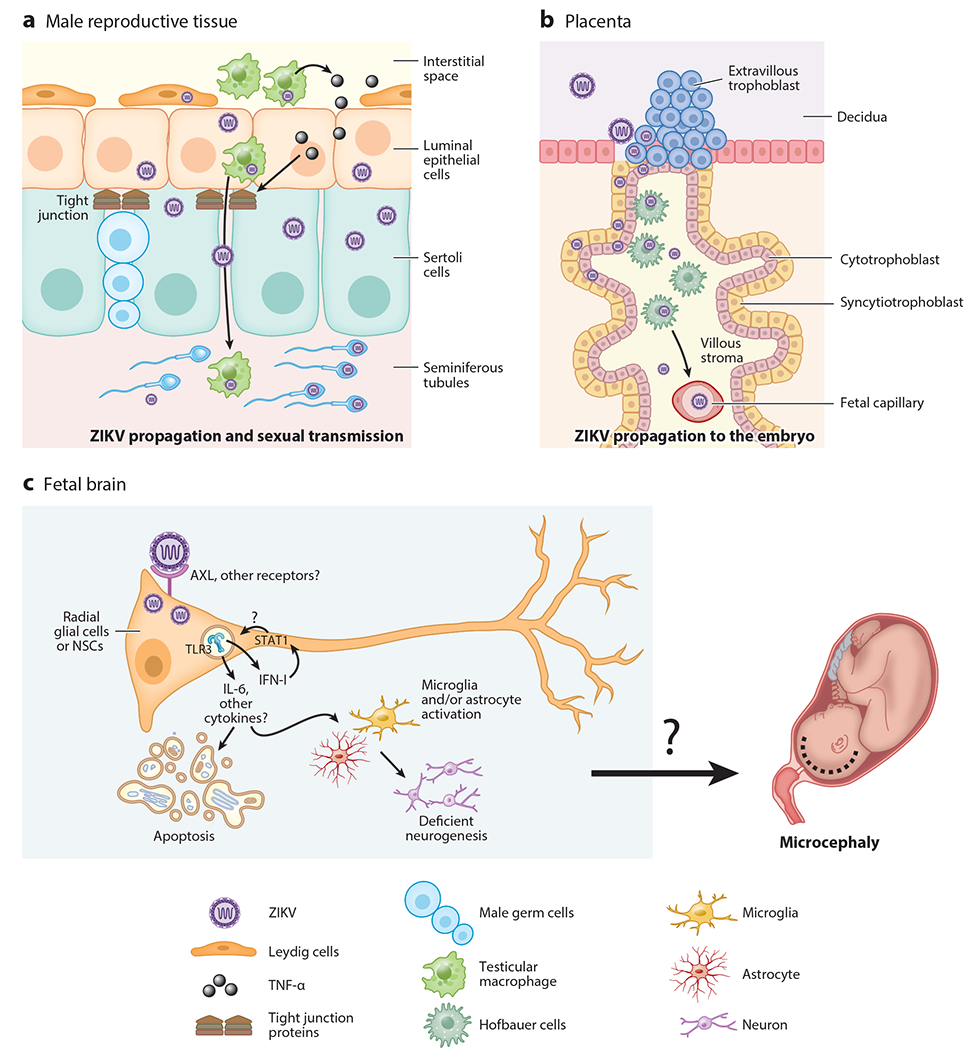
Zika virus (ZIKV) pathogenesis and its target tissues. ZIKV infects and persists in the target tissues, causing its characteristic pathology. (*a*) ZIKV can travel through the male reproductive tract, within infected immune cells. Once the virus reaches the blood testicular barrier, it infects Leydig cells and activates resident macrophages. This leads to the induction of inflammatory mediators such as tumor necrosis factor alpha (TNF-α) that can activate the epithelial barrier and promote the disruption of the tight junctions. ZIKV can also infects Sertoli cells, spermatogonia, primary spermatocytes, and spermatozoa and can infect and replicate in the sperm. The virus can be sexually transmitted via these cell types. (*b*) In pregnant women, ZIKV can infect the placenta, where it breaks the decidua and chorionic villi and also infects endothelial cells, Hofbauer cells, and cytotrophoblasts, among others. ZIKV can cross the maternal-fetal blood barrier and infect the embryo. (*c*) In the fetus, ZIKV efficiently infects neural stem cells (NSCs) during the first trimester of gestation. AXL has been proposed as one important receptor; however, this has not been confirmed in vivo, and a dominant receptor for ZIKV has not been found yet. Proinflammatory cytokines, including interleukin 6 (IL-6) and type I interferons (IFN-I), are induced via Toll-like receptor 3 (TLR3) and other pattern recognition receptors upon ZIKV infection and can promote tissue damage by causing apoptosis of neural stem cells and reducing neurogenesis. Together, these can contribute to pathology, potentially including microcephaly.

An interesting aspect of ZIKV is that it can be transmitted through sexual contact and may persist in the male reproductive tract (103). The testis is a privileged immunological environment. Here, the developing spermatozoa are protected from autoimmune attack by a physical hematotesticular barrier formed by tight junctions between adjacent Sertoli cells that prevent the entry of immunoglobulins as well as through the suppression of inflammatory responses (104, 105). Some reports indicated that ZIKV RNA has been detected in the semen of men for several months after onset of infection (106). Similarly, ZIKV RNA can be found in semen during the clinically symptomatic phase of the infection (107). The male reproductive tract has special characteristics that allow pathogen persistence and, therefore, the dispersion of viruses via sexual transmission (108). Studies in immunodeficient mice have shown that ZIKV has the ability to infect a wide variety of cells in male reproductive tissue, including luminal epithelial cells, Sertoli cells, Leydig cells, spermatogonia, primary spermatocytes, and spermatogonia peritubular myoid cells (109, 110) (Figure 3a). In humans, several studies have shown that ZIKV can also infect somatic and germ cells and is considered the cause of transmission to the mother and subsequent damage to the embryo (111–114). In some animal models, in the female reproductive tract, ZIKV infection was observed in the cervix and vagina, possibly due to viral evasion of immune responses to enhance ZIKV replication (115, 116). These studies provide further evidence that ZIKV can infect vaginal tissue during pregnancy and infect the embryo (117). Importantly, experiments performed in a mouse model showed that subcutaneous or intravaginal infection with ZIKV can protect from a secondary intravaginal challenge due to presence of high levels of neutralizing antibodies and ZIKV specific T cells (118). Passive transfer of specific antibodies or T cells also reduced intravaginal infection (118), suggesting that an adaptive immune response can protect against sexually transmitted vaginal infection.

During pregnancy, hormones can modulate the immune system as a mechanism of protection for the embryo. Hormones promote the generation of tolerogenic DCs, decrease monocyte and macrophage activity, and inhibit the recruitment of natural killer, T, and B cells to the maternal-fetal barrier (119–121). ZIKV-infected pregnant mice exhibit increased levels of macrophages and DCs in the uterus with a tolerogenic phenotype, with reduced frequencies of T cells and the presence of high viral RNA levels (122). While this tolerogenic environment may be beneficial for the embryo, it might concomitantly confer an advantage for virus dissemination. This may allow ZIKV to migrate through the placental barrier where it breaks the decidua and chorionic villi of the placenta, infecting placental cells such as cytotrophoblasts, endothelial cells, syncytiotrophoblasts, mesenchymal cells, fibroblasts, Hofbauer cells, macrophages, and decidual cells (117, 123–126) (Figure 3b). ZIKV infection persists in the placenta for a long time without the presence of symptoms and, thus, continues to infect the embryo (123, 125, 127).

Studies in ZIKV-infected pregnant nonhuman primates have shown inflammatory responses in the fetus and in the maternal-fetal interphase and result in fetal brain infection and neuroinflammation (21). Specifically, expression of IL-6 in the fetal brain can be associated with defects in radial glia and deficient neurogenesis and can also lead to fetal death (21, 128–130) (Figure 3c). Regarding the effects of IFN-I, studies carried out in mice showed detrimental effects on pregnancy due to activation of apoptosis of endothelial cells and trophoblasts. This led to suppression of placenta development, which damaged the maternal-fetal blood barrier and led to hypoxia and, ultimately, fetal death (131, 132). In addition, ZIKV infection of human neural stem/progenitor cells appeared to result in overactivation of innate pathways, including IRF7, TLR3, and other ISGs, while reducing expression of genes important in neurogenesis; this suggests a link between immune overactivation and inhibition of neurogenesis. Indeed, pharmacological inhibition of STAT1 reduced the detrimental effects observed in neurogenesis caused by ZIKV (133) (Figure 3c). Together, these studies suggest that inflammation and the IFN-I-mediated response could potentially be associated with complications during pregnancy; therefore, targeting the detrimental effects of IFN-I via therapeutics while preserving its antiviral roles could help prevent pathology during pregnancy.

## 4. ROLE OF THE HOST UBIQUITIN SYSTEM IN ZIKV REPLICATION, TISSUE TROPISM, AND PATHOGENESIS

Why does ZIKV replicate so efficiently in the reproductive and fetal tissue, leading to embryonic damage? So far, the evidence suggests that there are multiple receptors for ZIKV that are not specifically expressed in the placenta and/or embryonic tissue; therefore, expression of a dominant receptor might not explain ZIKV’s characteristic tropism. However, recent studies have shed some light on an alternative and novel mechanism of virus tropism. Since viruses need host cell machinery to replicate, the expression or activity of specific factors in a cell-type- or tissue-specific manner could provide a more beneficial environment for replication. For instance, the host ubiquitin system, a posttranslational process most well known for its function in targeting proteins for proteasomal degradation, can be utilized by flaviviruses to replicate efficiently in both a proteasome-dependent and -independent manner and may involve degradative and nondegradative mechanisms, ultimately causing pathogenesis (134–137). Proteasomal activity has been associated with more efficient replication of DENV in mosquito systems (138, 139) but has not yet been replicated for ZIKV There are also indirect mechanisms by which the ubiquitin system can promote viral replication and pathogenesis, including degradative and nondegradative negative regulation of innate antiviral pathways as well as viral antagonism of the IFN response (134, 140, 141).

**Figure 4:**
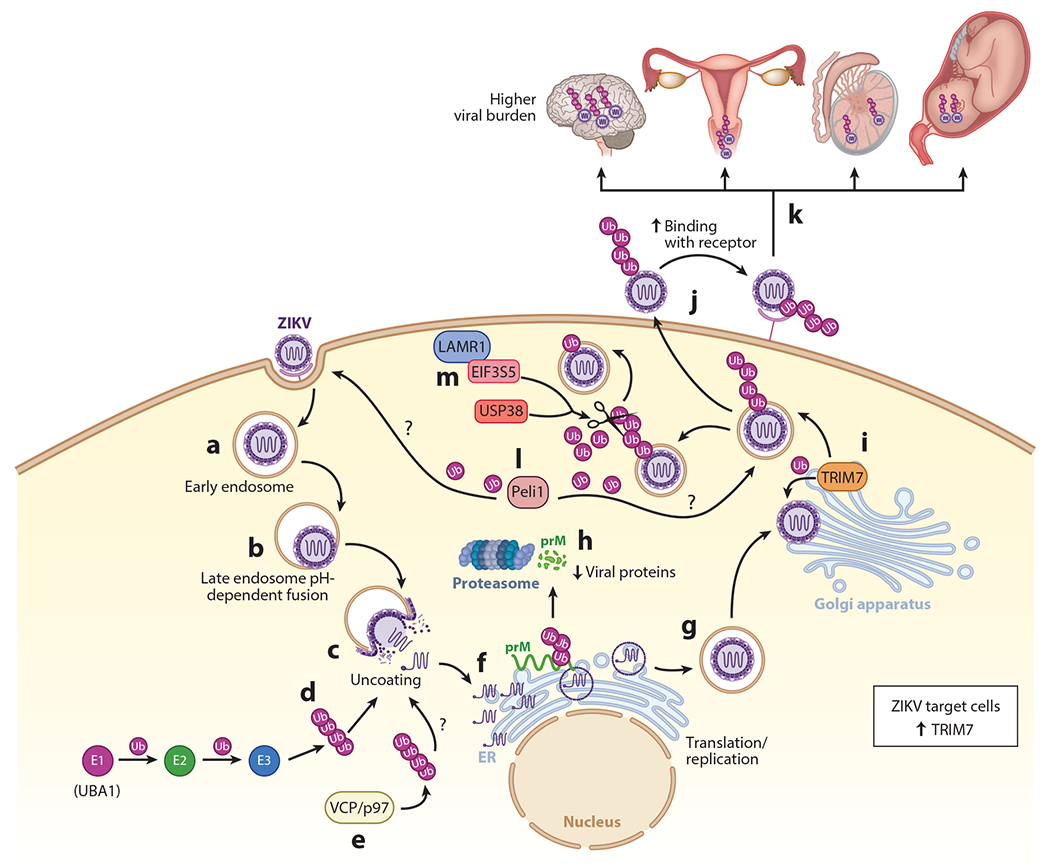
The role of the ubiquitin system in Zika virus (ZIKV) pathogenesis and tissue tropism. (*a*) After ZIKV attachment to cell receptors, the virus enters via endosomes. (*b*) Acidification in the endosome allows fusion of the envelope with the endosomal membrane. (*c*) The virus is then released in the cytoplasm after uncoating. (*d*) This step can be blocked with inhibitors of the E1-activating enzyme (UBA1) required for ubiquitination (Ub) of proteins, suggesting uncoating requires an ubiquitination step. How exactly ubiquitination mediates the uncoating step is unknown. (*e*) The ATPase valosin-containing protein p97 (VCP/p97) has been proposed to be involved in uncoating and speculated to do so by extracting an ubiquitinated protein during the uncoating process. (*f*) After uncoating, the viral RNA is replicated and transcribed in the endoplasmic reticulum (ER). The polypeptide is cleaved into the different viral proteins. (*g*) After virus assembly, which starts in the ER, the immature virion travels through the Golgi, where precursor membrane (prM) is cleaved to mature the virion and it continues to exit the cell. (*h*) prM protein of ZIKV is ubiquitinated on K6, resulting in proteasomal degradation. (*i*) The envelope protein of ZIKV is ubiquitinated by tripartite motif containing 7 (TRIM7) on K38 and K281, presumably around the Golgi or Golgi-associated membranes. (*j*) ZIKV-infectious virions released outside the cell containing a proportion of the ubiquitinated envelope can attach to receptors more efficiently, and provide a determinant of tissue tropism, by enhancing virus entry. (*k*) This further leads to increased viral loads in target tissues (brain, reproductive tissues, and embryo). (*l*) The E3-ubiquitin ligase Pellino 1 (Peli1) promotes entry and replication, but the viral target protein of ubiquitination is not known. (*m*) The host deubiquitinates ubiquitin-specific peptidase 38 (USP38) and eukaryotic translation initiation factor 3 subunit 5 (EIF3S5), which interact with laminin receptor 1 (LAMR1) to reduce the ubiquitination levels of the envelope protein and restrict ZIKV replication.

A recent report proposed a mechanism in which the ubiquitination process promotes virus attachment to host receptors. This study identified nondegradative K63-linked polyubiquitination on two lysine (K) residues of the ZIKV envelope protein (E-K38 and E-K281). Ubiquitination on the E-K38 residue, which is conserved in close relatives such as DENV, WNV, and YFV, is present in a small proportion of infectious virions and promotes stronger interactions with cell receptors (137). Importantly, the proportion of ubiquitinated envelope present in ZIKV infectious particles depends on which cell types are used to expand virus stocks. For example, placenta-derived JEG3 cells produced a higher ratio of ubiquitinated versus nonubiquitinated ZIKV as compared with viruses grown in African green monkey Vero cells, which are commonly used to expand viruses. This was confirmed using recombinant infectious mutant viruses that lack ubiquitination on the E-K38 site (E-K38R) and immunoprecipitation assays (137). The ZIKV E-K38R mutant virus replicated at a significantly lower rate in placental and brain cells as compared with WT virus in tissue culture. Similarly, the replication rate of the E-K38R virus was also lower as compared with WT ZIKV in mouse tissues after infection in vivo, with the largest differences observed in testes, uterus, eye, and brain, and caused reduced weight loss and death (137). This suggested that a factor expressed in specific tissues and/or cell types may provide ZIKV a more advantageous environment for replication. Tripartite motif (TRIM) containing 7 (TRIM7), a member of the E3-ubiquitin ligase TRIM family of proteins, was identified as this cellular factor that ubiquitinates the envelope protein of ZIKV. TRIM7 was found to promote ubiquitination of the ZIKV envelope protein in cells and in vitro, and TRIM7 knockout cells showed decreased virus replication. In addition, infection of *Trim7*^−/−^ mice with ZIKV showed reduced viral replication as compared with WT mice, especially in ZIKV-targeted tissues such as brain and reproductive tissues (137). It was also observed that the ubiquitination of the K38 residue improves binding to the TIM-1 cell receptor, leading to enhanced virus entry and replication (137) (Figure 4). Interestingly, the E-K38R mutant virus replicated similarly to WT virus in live mosquitoes, suggesting that ubiquitination on the K38 residue of the ZIKV envelope contributes to virus replication and pathogenesis in the mammalian host but not in the mosquito host and is one determinant of tissue tropism (137). This study focused on the role of the K38 site, but the role of the K281 in tissue tropism or perhaps the embryo remains to be investigated.

Additional studies have validated the ubiquitination of the ZIKV envelope protein and provide further support for its role in enhancing virus replication. It was found that a host deubiquitinase can reduce levels of the ubiquitinated envelope as an antiviral mechanism. Overexpression of USP38 (ubiquitin-specific peptidase 38) in HeLa cells, which is also known to regulate inflammation and histone modification and can inhibit IFN-I signaling during viral infection (142, 143), reduced ZIKV infection. In contrast, USP38 silencing using siRNA resulted in increased ZIKV replication. Also, specific binding to the envelope protein led to reduced ubiquitination, as evidenced by immunoprecipitation (144). Similarly, another host protein found to inhibit ZIKV replication via a mechanism involving deubiquitination of the envelope was laminin receptor 1 (LAMR1), a protein usually associated with cellular membranes that has multiple functions including regulating cell migration, differentiation, and other functions (145). It was found that eukaryotic translation initiation factor 3 subunit 5 (EIF3S5) deubiquitinase is recruited by LAMR1 to interact with and deubiquitinate the ZIKV envelope protein (146). Although these two studies provide further evidence that the ZIKV envelope protein is ubiquitinated, and that this ubiquitination is important in promoting virus replication, the researchers did not examine whether the effects of LAMR1 or USP38 affect the levels of the ubiquitinated envelope contained in the released infectious virion. These findings provide insights into the mechanisms by which ubiquitination restricts ZIKV infection through attenuation of protein E ubiquitination, suggesting that ubiquitination of the envelope can be used for the design of potential drugs for ZIKV infection.

TRIM7 is not the only E3-ubiquitin ligase used by ZIKV to promote viral replication. Pellino 1 (Peli1), an E3-ubiquitin ligase that is expressed in many cell types, has also been identified as promoting viral replication in ZIKV. Peli1 is responsible for regulating the production of proinflammatory cytokines activated by PRRs and is attributed to the modulation of necroptosis and apoptosis (147, 148). Expression of Peli1 increases significantly during ZIKV infection and can also promote vertical transmission, thereby mediating inflammation and cell death in placental and neuronal cells (148). This study proposed that Peli1 is involved in ZIKV replication at different stages of the replication cycle including cell attachment, entry, and viral translation, although the precise mechanism was not elucidated. In addition, the study did not address whether Peli1 ubiquitinates a viral protein or whether the effects observed may be indirect due to other functions of Peli1. It will be interesting to see whether Peli1 may play a redundant role to TRIM7 in ubiquitination of the ZIKV envelope protein.

Another Zika viral protein that is ubiquitinated is prM, but this ubiquitination results in degradation. This was demonstrated by coexpressing the envelope protein with the mutant protein prM-K6R deficient in ubiquitination, which resulted in decreased secretion of viral proteins compared with WT prM (149) (Figure 4).

Ubiquitination of viral proteins also appears to play a role in other stages of the replication cycle. For example, DENV capsid protein is degraded by the proteasome after viral internalization, but this degradation is not necessary to release the viral genome to allow viral RNA translation. However, pharmaceutical inhibition of the E1-ubiquitin-activating enzyme UBA1 blocked genome uncoating and inhibition of viral RNA translation (135). Although K-to-R mutants were used, ubiquitination of a specific lysine residue on capsid was not conclusively identified (135). Although these findings have so far not been fully confirmed for ZIKV, similar findings were observed for YFV using the UBA1 inhibitor. In addition, this study found that chemical inhibition of the valosin-containing protein VCP/p97, a cellular ATPase that associates with ubiquitinated proteins (150), reduced expression of the YFV reporter after entry but before translation of the incoming genome (151). This suggests that flavivirus uncoating may require a general mechanism of ubiquitination (Figure 4). It has also been proposed that free, noncovalently attached ubiquitin may be present in ZIKV particles that may promote uncoating (152). Together, these reports suggest that within the functions of the ubiquitin system there is also a nondegrading function of virus ubiquitination necessary for the replication of flaviviruses during the uncoating step. Together, these studies demonstrate the ability of ZIKV to hijack the cellular ubiquitination machinery to promote viral replication and pathogenesis.

## 5. CONCLUSIONS AND FUTURE DIRECTIONS

ZIKV continues to circulate in tropical and subtropical regions with the potential of reemerging epidemics once the COVID-19 pandemic dissipates. This continues to pose a significant risk, especially for pregnant women. One particular area that needs increased efforts is a better understanding of how ZIKV may dysregulate the immune response to cause detrimental inflammatory effects during pregnancy and specifically to the embryo. In addition, since the virus requires host factors for its replication, further research is needed to understand the mechanism involved in ZIKV pathogenesis and how it uses cellular machinery for viral replication and dissemination. One area that requires attention is a better understanding of how ZIKV enters its target cells and tissues. The identification of a major receptor should be a priority; however, since it is possible that there is no dominant receptor, studies should focus on other host factors that provide a beneficial environment for ZIKV to replicate. Furthermore, future studies need to carefully consider which cells are used to expand virus stocks. Many studies have used ZIKV produced in mosquito cells (C636) or green monkey kidney epithelial cells (Vero) instead of using more physiologically relevant cells (e.g., cells of placental origin). As we discussed above, viruses grown in mosquito cells could lack ubiquitination on the envelope protein, which may limit the identification of entry factors. Since ubiquitin chains can directly interact with the TIM-1 receptor (137), another future area of research is the identification of ubiquitin-binding domains potentially present on the putative receptors of factors involved in virus entry and uncoating. This could identify specific regions on the receptors to target pharmacologically in order to break interactions with the ubiquitinated ZIKV envelope.

Importantly, since specific antibodies against K63-linked polyubiquitin chains can partially neutralize ZIKV and reduce infectivity in a mouse model (137), another important field to explore is the development of antibodies directed at ubiquitin for ZIKV neutralization. Further studies are needed to characterize the positive and negative effects of this antibody in mice pregnancy models, before they can be used as therapy against ZIKV.

In this review, we provide an overview of the components involved in the interaction between ZIKV proteins and host factors as well as the mechanism used by ZIKV to activate or evade the immune response. Overall, the identification of host-specific factors that are commonly required for replication of different viruses, especially closely related flaviviruses, could provide novel targets for broad-spectrum antivirals.

## Abbreviations

ZIKV: Zika virus
NS: nonstructural
fNSCs: fetal neural stem cells
DENV: Dengue virus
YFV: yellow fever virus
WNV: West Nile virus
prM: precursor membrane
TRIM7: tripartite motif containing 7
USP38: ubiquitin-specific peptidase 38
LAMR1: laminin receptor 1
EIF3S5: eukaryotic translation initiation factor 3 subunit 5
Peli1: Pellino 1

## References

[1] Sirohi D, Chen Z, Sun L, Klose T, Pierson TC, 2016. The 3.8 Å resolution cryo-EM structure of Zika virus. Science 352:467–70 27033547 10.1126/science.aaf5316 PMC4845755

[2] Gutierrez-Bugallo G, Piedra LA, Rodriguez M, Bisset JA, Lourenco-de-Oliveira R, 2019. Vector-borne transmission and evolution of Zika virus. Nat. Ecol. Evol 3:561–69 30886369 10.1038/s41559-019-0836-z PMC8900209

[3] Pletnev AG, Maximova OA, Liu G, Kenney H, Nagata BM, 2021. Epididymal epithelium propels early sexual transmission of Zika virus in the absence of interferon signaling. Nat. Commun 12:2469 33927207 10.1038/s41467-021-22729-5 PMC8084954

[4] Miner JJ. 2017. Congenital Zika virus infection: more than just microcephaly. Sci. Transl. Med 9:8195 10.1126/scitranslmed.aan8195 28592568

[5] Dick GW, Kitchen SF, Haddow AJ. 1952. Zika virus. I. Isolations and serological specificity. Trans. R. Soc. Trop. Med. Hyg 46:509–20 12995440 10.1016/0035-9203(52)90042-4

[6] Simpson DI. 1964. Zika Virus infection in man. Trans. R. Soc. Trop. Med. Hyg 58:335–38 14175744

[7] Moore DL, Causey OR, Carey DE, Reddy S, Cooke AR, 1975. Arthropod-borne viral infections of man in Nigeria, 1964–1970. Ann. Trop. Med. Parasitol 69:49–64 1124969 10.1080/00034983.1975.11686983

[8] Olson JG, Ksiazek TG, Suhandiman T. 1981. Zika virus, a cause of fever in Central Java, Indonesia. Trans. R. Soc. Trop. Med. Hyg 75:389–93 6275577 10.1016/0035-9203(81)90100-0

[9] Petersen LR, Jamieson DJ, Honein MA. 2016. Zika virus. N. Engl. J. Med 375:294–95 10.1056/NEJMc1606769 27355409

[10] Duffy MR, Chen TH, Hancock WT, Powers AM, Kool JL, 2009. Zika virus outbreak on Yap Island, Federated States of Micronesia. N. Engl. J. Med 360:2536–43 19516034 10.1056/NEJMoa0805715

[11] Ribeiro IG, Andrade MR, Silva JM, Silva ZM, Costa MAO, 2018. Microcephaly in Piauí, Brazil: descriptive study during the Zika virus epidemic, 2015–2016. Epidemiol. Serv. Saúde 27:e20163692 29412347 10.5123/S1679-49742018000100002

[12] World Health Organ. (WHO). 2022. Countries and territories with current or previous Zika virus transmission. Map, WHO Health Emerg. Progr., Geneva https://cdn.who.int/media/docs/default-source/documents/emergencies/zika/map-of-countries_with_zika_transmission_feb2022.pdf

[13] Inst. Nac. de Salud de Colomb. (INS). 2021. Reporte boletin epidemiologico semana 43. Rep, INS, Bogotá, Colombia

[14] Dir. Gen. Epidemiol. (DGE). 2021. Boletin epidemiológico: sistema nacionalde vigilancia epidemológica semana 41. Rep., Secr de Salud de Méx., Mexico City

[15] Carod-Artal FJ. 2018. Neurological complications of Zika virus infection. Expert Rev. Anti. Infect. Ther 16:399–410 29668332 10.1080/14787210.2018.1466702

[16] Reynolds MR, Jones AM, Petersen EE, Lee EH, Rice ME, 2017. Vital signs: update on Zika virus–associated birth defects and evaluation of all U.S. infants with congenital Zika virus exposure—U.S. Zika Pregnancy Registry, 2016. MMWR Morb. Mortal. Wkly. Rep 66:366–73 28384133 10.15585/mmwr.mm6613e1 PMC5657905

[17] Smoots AN, Olson SM, Cragan J, Delaney A, Roth NM, 2020. Population-based surveillance for birth defects potentially related to Zika virus infection—22 states and territories, January 2016–June 2017. MMWR Morb. Mortal. Wkly. Rep 69:67–71 31971935 10.15585/mmwr.mm6903a3 PMC7367037

[18] World Health Organ. (WHO). 2018. Zika virus. Factsheet, WHO, Geneva

[19] Paixao ES, Cardim LL, Costa MCN, Brickley EB, de Carvalho-Sauer RCO, 2022. Mortality from congenital Zika syndrome—nationwide cohort study in Brazil. N. Engl. J. Med 386:757–67 35196428 10.1056/NEJMoa2101195 PMC7612437

[20] Caine EA, Scheaffer SM, Arora N, Zaitsev K, Artyomov MN, 2019. Interferon lambda protects the female reproductive tract against Zika virus infection. Nat. Commun 10:280 30655513 10.1038/s41467-018-07993-2 PMC6336786

[21] Casazza RL, Lazear HM, Miner JJ. 2020. Protective and pathogenic effects of interferon signaling during pregnancy. Viral Immunol. 33:3–11 31545139 10.1089/vim.2019.0076 PMC6978785

[22] Serman TM, Gack MU. 2019. Evasion of innate and intrinsic antiviral pathways by the Zika virus. Viruses 11:970 31652496 10.3390/v11100970 PMC6833475

[23] Estevez-Herrera J, Perez-Yanes S, Cabrera-Rodriguez R, Marquez-Arce D, Trujillo-Gonzalez R, 2021. Zika Virus pathogenesis: a battle for immune evasion. Vaccines 9:294 33810028 10.3390/vaccines9030294 PMC8005041

[24] Coldbeck-Shackley RC, Eyre NS, Beard MR. 2020. The molecular interactions of ZIKV and DENV with the type-I IFN response. Vaccines 8:530 32937990 10.3390/vaccines8030530 PMC7565347

[25] Pardy RD, Valbon SF, Richer MJ. 2019. Running interference: interplay between Zika virus and the host interferon response. Cytokine 119:7–15 30856603 10.1016/j.cyto.2019.02.009

[26] Choi Y, Bowman JW, Jung JU. 2018. Autophagy during viral infection—a double-edged sword. Nat. Rev. Microbiol 16:341–54 29556036 10.1038/s41579-018-0003-6 PMC6907743

[27] Deretic V, Saitoh T, Akira S. 2013. Autophagy in infection, inflammation and immunity. Nat. Rev. Immunol 13:722–37 24064518 10.1038/nri3532 PMC5340150

[28] Ke PY. 2021. Autophagy and antiviral defense. IUBMB Life 74:317–38 34859938 10.1002/iub.2582

[29] Munz C. 2009. Enhancing immunity through autophagy. Annu. Rev. Immunol 27:423–49 19105657 10.1146/annurev.immunol.021908.132537

[30] Peng H, Liu B, Yves TD, He Y, Wang S, 2018. Zika virus induces autophagy in human umbilical vein endothelial cells. Viruses 10:259 29762492 10.3390/v10050259 PMC5977252

[31] Klaitong P, Smith DR. 2021. Roles of non-structural protein 4A in flavivirus infection. Viruses 13:2077 34696510 10.3390/v13102077 PMC8538649

[32] Liang Q, Luo Z, Zeng J, Chen W, Foo SS, 2016. Zika Virus NS4A and NS4B proteins deregulate Akt-mTOR signaling in human fetal neural stem cells to inhibit neurogenesis and induce autophagy. Cell Stem Cell 19:663–71 27524440 10.1016/j.stem.2016.07.019 PMC5144538

[33] Franke TF. 2008. PI3K/Akt: getting it right matters. Oncogene 27:6473–88 18955974 10.1038/onc.2008.313

[34] Cao B, Parnell LA, Diamond MS, Mysorekar IU. 2017. Inhibition of autophagy limits vertical transmission of Zika virus in pregnant mice. J. Exp. Med 214:2303–13 28694387 10.1084/jem.20170957 PMC5551583

[35] Hamel R, Dejarnac O, Wichit S, Ekchariyawat P, Neyret A, 2015. Biology of Zika virus infection in human skin cells. J. Virol 89:8880–96 26085147 10.1128/JVI.00354-15 PMC4524089

[36] Han X, Wang J, Yang Y, Qu S, Wan F, 2021. Zika virus infection induced apoptosis by modulating the recruitment and activation of pro-apoptotic protein Bax. J. Virol 95:e01445–20 10.1128/JVI.01445-20 PMC8103684 33536166

[37] Tang H, Hammack C, Ogden SC, Wen Z, Qian X, 2016. Zika Virus infects human cortical neural progenitors and attenuates their growth. Cell Stem Cell 18:587–90 26952870 10.1016/j.stem.2016.02.016 PMC5299540

[38] Souza BS, Sampaio GL, Pereira CS, Campos GS, Sardi SI, 2016. Zika virus infection induces mitosis abnormalities and apoptotic cell death of human neural progenitor cells. Sci. Rep 6:39775 28008958 10.1038/srep39775 PMC5180086

[39] Liu J, Li Q, Li X, Qiu Z, Li A, 2018. Zika virus envelope protein induces G2/M cell cycle arrest and apoptosis via an intrinsic cell death signaling pathway in neuroendocrine PC12 cells. Int. J. Biol. Sci 14:1099–108 29989100 10.7150/ijbs.26400 PMC6036729

[40] Guo Z, Li Y, Ding SW. 2019. Small RNA-based antimicrobial immunity. Nat. Rev. Immunol 19:31–44 30301972 10.1038/s41577-018-0071-x

[41] Maillard PV, Ciaudo C, Marchais A, Li Y, Jay F, 2013. Antiviral RNA interference in mammalian cells. Science 342:235–38 24115438 10.1126/science.1241930 PMC3853215

[42] Cullen BR, Cherry S, tenOever BR. 2013. Is RNA interference a physiologically relevant innate antiviral immune response in mammals? Cell Host Microbe 14:374–78 24139396 10.1016/j.chom.2013.09.011

[43] Ding SW. 2010. RNA-based antiviral immunity. Nat. Rev. Immunol 10:632–44 20706278 10.1038/nri2824

[44] Poirier EZ, Buck MD, Chakravarty P, Carvalho J, Frederico B, 2021. An isoform of Dicer protects mammalian stem cells against multiple RNA viruses. Science 373:231–36 34244417 10.1126/science.abg2264 PMC7611482

[45] Zeng J, Dong S, Luo Z, Xie X, Fu B, 2020. The Zika virus capsid disrupts corticogenesis by suppressing Dicer activity and miRNA biogenesis. Cell Stem Cell 27:618–32.e9 32763144 10.1016/j.stem.2020.07.012 PMC7541724

[46] Xu YP, Qiu Y, Zhang B, Chen G, Chen Q, 2019. Zika virus infection induces RNAi-mediated antiviral immunity in human neural progenitors and brain organoids. Cell Res. 29:265–73 30814679 10.1038/s41422-019-0152-9 PMC6461993

[47] Zeng J, Luo Z, Dong S, Xie X, Liang X, 2021. Functional mapping of AGO-associated Zika virus-derived small interfering RNAs in neural stem cells. Front. Cell. Infect. Microbiol 11:628887 33718276 10.3389/fcimb.2021.628887 PMC7946837

[48] Schoggins JW. 2019. Interferon-stimulated genes: What do they all do? Annu. Rev. Virol 6:567–84 31283436 10.1146/annurev-virology-092818-015756

[49] Suthar MS, Aguirre S, Fernandez-Sesma A. 2013. Innate immune sensing of flaviviruses. PLOS Pathog. 9:e1003541 24068919 10.1371/journal.ppat.1003541 PMC3771895

[50] Valdes Lopez JF, Velilla PA, Urcuqui-Inchima S. 2019. Chikungunya virus and Zika virus, two different viruses examined with a common aim: role of pattern recognition receptors on the inflammatory response. J. Interferon Cytokine Res 39:507–21 31090481 10.1089/jir.2019.0058

[51] Stetson DB, Medzhitov R. 2006. Type I interferons in host defense. Immunity 25:373–81 16979569 10.1016/j.immuni.2006.08.007

[52] Levy DE, Marie IJ, Durbin JE. 2011. Induction and function of type I and III interferon in response to viral infection. Curr. Opin. Virol 1:476–86 22323926 10.1016/j.coviro.2011.11.001 PMC3272644

[53] Matsumoto M, Seya T. 2008. TLR3: interferon induction by double-stranded RNA including poly(I:C). Adv. Drug. Deliv. Rev 60:805–12 18262679 10.1016/j.addr.2007.11.005

[54] Seth RB, Sun L, Ea CK, Chen ZJ. 2005. Identification and characterization of MAVS, a mitochondrial antiviral signaling protein that activates NF-κB and IRF3. Cell 122:669–82 16125763 10.1016/j.cell.2005.08.012

[55] Loo YM, Gale M Jr. 2011. Immune signaling by RIG-I-like receptors. Immunity 34:680–92 21616437 10.1016/j.immuni.2011.05.003 PMC3177755

[56] Webb LG, Fernandez-Sesma A. 2022. RNA viruses and the cGAS-STING pathway: reframing our understanding of innate immune sensing. Curr. Opin. Virol 53:101206 35180533 10.1016/j.coviro.2022.101206

[57] Aguirre S, Luthra P, Sanchez-Aparicio MT, Maestre AM, Patel J, 2017. Dengue virus NS2B protein targets cGAS for degradation and prevents mitochondrial DNA sensing during infection. Nat. Microbiol 2:17037 28346446 10.1038/nmicrobiol.2017.37 PMC7457382

[58] Zheng Y, Liu Q, Wu Y, Ma L, Zhang Z, 2018. Zika virus elicits inflammation to evade antiviral response by cleaving cGAS via NS1-caspase-1 axis. EMBO J. 37:e99347 30065070 10.15252/embj.201899347 PMC6138430

[59] Rossi SL, Tesh RB, Azar SR, Muruato AE, Hanley KA, 2016. Characterization of a novel murine model to study Zika virus. Am. J. Trop. Med. Hyg 94:1362–69 27022155 10.4269/ajtmh.16-0111 PMC4889758

[60] Xia H, Luo H, Shan C, Muruato AE, Nunes BTD, 2018. An evolutionary NS1 mutation enhances Zika virus evasion of host interferon induction. Nat. Commun 9:414 29379028 10.1038/s41467-017-02816-2 PMC5788864

[61] Dang J, Tiwari SK, Lichinchi G, Qin Y, Patil VS, 2016. Zika virus depletes neural progenitors in human cerebral organoids through activation of the innate immune receptor TLR3. Cell Stem Cell 19:258–65 27162029 10.1016/j.stem.2016.04.014 PMC5116380

[62] Vanwalscappel B, Tada T, Landau NR. 2018. Toll-like receptor agonist R848 blocks Zika virus replication by inducing the antiviral protein viperin. Virology 522:199–208 30036788 10.1016/j.virol.2018.07.014 PMC6130814

[63] Bowen JR, Quicke KM, Maddur MS, O’Neal JT, McDonald CE, 2017. Zika virus antagonizes type I interferon responses during infection of human dendritic cells. PLOS Pathog. 13:e1006164 28152048 10.1371/journal.ppat.1006164 PMC5289613

[64] Ma J, Ketkar H, Geng T, Lo E, Wang L, 2018. Zika virus non-structural protein 4A blocks the RLR-MAVS signaling. Front. Microbiol 9:1350 29988497 10.3389/fmicb.2018.01350 PMC6026624

[65] Esser-Nobis K, Aarreberg LD, Roby JA, Fairgrieve MR, Green R, Gale M Jr. 2019. Comparative analysis of African and Asian lineage-derived Zika virus strains reveals differences in activation of and sensitivity to antiviral innate immunity. J. Virol 93:e00640–19 31019057 10.1128/JVI.00640-19 PMC6580957

[66] Hertzog J, Dias AG Junior, Rigby RE, Donald CL, Mayer A, 2018. Infection with a Brazilian isolate of Zika virus generates RIG-I stimulatory RNA and the viral NS5 protein blocks type I IFN induction and signaling. Eur. J. Immunol 48:1120–36 29572905 10.1002/eji.201847483 PMC6055886

[67] Hage A, Bharaj P, van Tol S, Giraldo MI, Gonzalez-Orozco M, 2022. The RNA helicase DHX16 recognizes specific viral RNA to trigger RIG-I-dependent innate antiviral immunity. Cell Rep. 38(10):110434 35263596 10.1016/j.celrep.2022.110434 PMC8903195

[68] Parisien JP, Lenoir JJ, Alvarado G, Horvath CM. 2022. The human STAT2 coiled-coil domain contains a degron for Zika virus interferon evasion. J. Virol 96:e0130121 34643427 10.1128/JVI.01301-21 PMC8754212

[69] Grant A, Ponia SS, Tripathi S, Balasubramaniam V, Miorin L, 2016. Zika virus targets human STAT2 to inhibit type I interferon signaling. Cell Host Microbe 19:882–90 27212660 10.1016/j.chom.2016.05.009 PMC4900918

[70] Wu Y, Liu Q, Zhou J, Xie W, Chen C, 2017. Zika virus evades interferon-mediated antiviral response through the co-operation of multiple nonstructural proteins in vitro. Cell Discov. 3:17006 28373913 10.1038/celldisc.2017.6 PMC5359216

[71] Shu J, Ma X, Zhang Y, Zou J, Yuan Z, Yi Z.2021. NS5-independent ablation of STAT2 by Zika virus to antagonize interferon signalling. Emerg. Microbes Infect 10:1609–25 34340648 10.1080/22221751.2021.1964384 PMC8366623

[72] Gorman MJ, Caine EA, Zaitsev K, Begley MC, Weger-Lucarelli J, 2018. An immunocompetent mouse model of Zika virus infection. Cell Host Microbe 23:672–85.e6 29746837 10.1016/j.chom.2018.04.003 PMC5953559

[73] Laurent-Rolle M, Morrison J. 2019. The role of NS5 protein in determination of host cell range for yellow fever virus. DNA Cell Biol. 38:1414–17 31633391 10.1089/dna.2019.5115

[74] Laurent-Rolle M, Morrison J, Rajsbaum R, Macleod JML, Pisanelli G, 2014. The interferon signaling antagonist function of yellow fever virus NS5 protein is activated by type I interferon. Cell Host Microbe 16:314–27 25211074 10.1016/j.chom.2014.07.015 PMC4176702

[75] Wang B, Thurmond S, Zhou K, Sanchez-Aparicio MT, Fang J, 2020. Structural basis for STAT2 suppression by flavivirus NS5. Nat. Struct. Mol. Biol 27:875–85 32778820 10.1038/s41594-020-0472-y PMC7554153

[76] Giraldo MI, Vargas-Cuartas O, Gallego-Gomez JC, Shi PY, Padilla-Sanabria L, 2018. K48-linked polyubiquitination of dengue virus NS1 protein inhibits its interaction with the viral partner NS4B. Virus Res. 246:1–11 29294313 10.1016/j.virusres.2017.12.013 PMC5811335

[77] Ding Q, Gaska JM, Douam F, Wei L, Kim D, 2018. Species-specific disruption of STING-dependent antiviral cellular defenses by the Zika virus NS2B3 protease. PNAS 115:E6310–18 29915078 10.1073/pnas.1803406115 PMC6142274

[78] Aguirre S, Maestre AM, Pagni S, Patel JR, Savage T, 2012. DENV inhibits type I IFN production in infected cells by cleaving human STING. PLOS Pathog. 8:e1002934 23055924 10.1371/journal.ppat.1002934 PMC3464218

[79] Yu CY, Chang TH, Liang JJ, Chiang RL, Lee YL, 2012. Dengue virus targets the adaptor protein MITA to subvert host innate immunity. PLOS Pathog. 8:e1002780 22761576 10.1371/journal.ppat.1002780 PMC3386177

[80] Fanunza E, Grandi N, Quartu M, Carletti F, Ermellino L, 2021. INMI1 Zika virus NS4B antagonizes the interferon signaling by suppressing STAT1 phosphorylation. Viruses 13:2448 34960717 10.3390/v13122448 PMC8705506

[81] Chan YK, Gack MU. 2016. A phosphomimetic-based mechanism of dengue virus to antagonize innate immunity. Nat. Immunol 17:523–30 26998762 10.1038/ni.3393 PMC4837045

[82] Riedl W, Acharya D, Lee JH, Liu G, Serman T, 2019. Zika virus NS3 mimics a cellular 14-3-3-binding motif to antagonize RIG-I- and MDA5-mediated innate immunity. Cell Host Microbe 26:493–503.e6 31600501 10.1016/j.chom.2019.09.012 PMC6922055

[83] Donald CL, Brennan B, Cumberworth SL, Rezelj VV, Clark JJ, 2016. Full genome sequence and sfRNA interferon antagonist activity of Zika virus from Recife, Brazil. PLOS Negl. Trop. Dis 10:e0005048 27706161 10.1371/journal.pntd.0005048 PMC5051680

[84] Manokaran G, Finol E, Wang C, Gunaratne J, Bahl J, 2015. Dengue subgenomic RNA binds TRIM25 to inhibit interferon expression for epidemiological fitness. Science 350:217–21 26138103 10.1126/science.aab3369 PMC4824004

[85] Winkler CW, Myers LM, Woods TA, Messer RJ, Carmody AB, 2017. Adaptive immune responses to Zika virus are important for controlling virus infection and preventing infection in brain and testes. J. Immunol 198:3526–35 28330900 10.4049/jimmunol.1601949 PMC5701572

[86] Pardy RD, Rajah MM, Condotta SA, Taylor NG, Sagan SM, Richer MJ. 2017. Analysis of the T cell response to Zika virus and identification of a novel CD8^+^ T cell epitope in immunocompetent mice. PLOS Pathog. 13:e1006184 28231312 10.1371/journal.ppat.1006184 PMC5322871

[87] Huang H, Li S, Zhang Y, Han X, Jia B, 2017. CD8^+^ T cell immune response in immunocompetent mice during Zika virus infection. J. Virol 91:e00900–17 28835502 10.1128/JVI.00900-17 PMC5660488

[88] Grifoni A, Pham J, Sidney J, O’Rourke PH, Paul S, 2017. Prior dengue virus exposure shapes T cell immunity to Zika virus in humans. J. Virol 91:e01469–17 28978707 10.1128/JVI.01469-17 PMC5709580

[89] Weiskopf D, Angelo MA, Sidney J, Peters B, Shresta S, Sette A. 2014. Immunodominance changes as a function of the infecting dengue virus serotype and primary versus secondary infection. J. Virol 88:11383–94 25056881 10.1128/JVI.01108-14 PMC4178794

[90] Weiskopf D, Angelo MA, Grifoni A, O’Rourke PH, Sidney J, 2016. HLA-DRB1 alleles are associated with different magnitudes of dengue virus-specific CD4^+^ T-cell responses. J. Infect. Dis 214:1117–24 27443615 10.1093/infdis/jiw309 PMC5021234

[91] El Sahly HM, Gorchakov R, Lai L, Natrajan MS, Patel SM, 2019. Clinical, virologic, and immunologic characteristics of Zika virus infection in a cohort of US patients: prolonged RNA detection in whole blood. Open Forum Infect. Dis 6:ofy352 30697574 10.1093/ofid/ofy352 PMC6343961

[92] Rogers TF, Goodwin EC, Briney B, Sok D, Beutler N, 2017. Zika virus activates de novo and cross-reactive memory B cell responses in dengue-experienced donors. Sci. Immunol 2:e6809 10.1126/sciimmunol.aan6809 PMC5892203 28821561

[93] Stettler K, Beltramello M, Espinosa DA, Graham V, Cassotta A, 2016. Specificity, cross-reactivity, and function of antibodies elicited by Zika virus infection. Science 353:823–26 27417494 10.1126/science.aaf8505

[94] Van Rompay KKA, Coffey LL, Kapoor T, Gazumyan A, Keesler RI, 2020. A combination of two human monoclonal antibodies limits fetal damage by Zika virus in macaques. PNAS 117:7981–89 32209664 10.1073/pnas.2000414117 PMC7149495

[95] Wang Y, Lobigs M, Lee E, Mullbacher A. 2003. CD8^+^ T cells mediate recovery and immunopathology in West Nile virus encephalitis. J. Virol 77:13323–34 14645588 10.1128/JVI.77.24.13323-13334.2003 PMC296062

[96] Manangeeswaran M, Ireland DD, Verthelyi D. 2016. Zika (PRVABC59) infection is associated with T cell infiltration and neurodegeneration in CNS of immunocompetent neonatal C57B1/6 mice. PLOS Pathog. 12:e1006004 27855206 10.1371/journal.ppat.1006004 PMC5113993

[97] Jurado KA, Yockey LJ, Wong PW, Lee S, Huttner AJ, Iwasaki A. 2018. Antiviral CD8 T cells induce Zika-virus-associated paralysis in mice. Nat. Microbiol 3:141–47 29158604 10.1038/s41564-017-0060-z PMC5780207

[98] Miner JJ, Diamond MS. 2017. Zika virus pathogenesis and tissue tropism. Cell Host Microbe 21:134–42 28182948 10.1016/j.chom.2017.01.004 PMC5328190

[99] Onorati M, Li Z, Liu F, Sousa AMM, Nakagawa N, 2016. Zika virus disrupts phospho-TBK1 localization and mitosis in human neuroepithelial stem cells and radial glia. Cell Rep. 16:2576–92 27568284 10.1016/j.celrep.2016.08.038 PMC5135012

[100] Xie S, Zhang H, Liang Z, Yang X, Cao R. 2021. AXL, an important host factor for DENV and ZIKV replication. Front. Cell. Infect. Microbiol 11:575346 33954117 10.3389/fcimb.2021.575346 PMC8092360

[101] Govero J, Esakky P, Scheaffer SM, Fernandez E, Drury A, 2016. Zika virus infection damages the testes in mice. Nature 540:438–42 27798603 10.1038/nature20556 PMC5432198

[102] Hastings AK, Yockey LJ, Jagger BW, Hwang J, Uraki R, 2017. TAM receptors are not required for Zika virus infection in mice. Cell Rep. 19:558–68 28423319 10.1016/j.celrep.2017.03.058 PMC5485843

[103] Coelho FC, Durovni B, Saraceni V, Lemos C, Codeco CT, 2016. Higher incidence of Zika in adult women than adult men in Rio de Janeiro suggests a significant contribution of sexual transmission from men to women. Int. J. Infect. Dis 51:128–32 27664930 10.1016/j.ijid.2016.08.023

[104] Fijak M, Meinhardt A. 2006. The testis in immune privilege. Immunol. Rev 213:66–81 16972897 10.1111/j.1600-065X.2006.00438.x

[105] Hedger MP. 2002. Macrophages and the immune responsiveness of the testis. J. Reprod. Immunol 57:19–34 12385831 10.1016/s0165-0378(02)00016-5

[106] Kurscheidt FA, Mesquita CSS, Damke G, Damke E, Carvalho A, 2019. Persistence and clinical relevance of Zika virus in the male genital tract. Nat. Rev. Urol 16:211–30 30696994 10.1038/s41585-019-0149-7

[107] D’Ortenzio E, Matheron S, Yazdanpanah Y, de Lamballerie X, Hubert B, 2016. Evidence of sexual transmission of Zika virus. N. Engl. J. Med 374:2195–98 27074370 10.1056/NEJMc1604449

[108] Dejucq-Rainsford N, Jegou B. 2004. Viruses in semen and male genital tissues—consequences for the reproductive system and therapeutic perspectives. Curr. Pharm. Des 10:557–75 14965339 10.2174/1381612043453225

[109] Clancy CS, Van Wettere AJ, Siddharthan V, Morrey JD, Julander JG. 2018. Comparative histopathologic lesions of the male reproductive tract during acute infection of Zika virus in AG129 and *Ifnar*^−/−^ mice. Am. J. Pathol 188:904–15 29378173 10.1016/j.ajpath.2017.12.019 PMC5955007

[110] Prow NA, Liu L, Nakayama E, Cooper TH, Yan K, 2018. A vaccinia-based single vector construct multi-pathogen vaccine protects against both Zika and chikungunya viruses. Nat. Commun 9:1230 29581442 10.1038/s41467-018-03662-6 PMC5964325

[111] Matusali G, Houzet L, Satie AP, Mahe D, Aubry F, 2018. Zika virus infects human testicular tissue and germ cells. J. Clin. Investig 128:4697–710 30063220 10.1172/JCI121735 PMC6159993

[112] Robinson CL, Chong ACN, Ashbrook AW, Jeng G, Jin J, 2018. Male germ cells support long-term propagation of Zika virus. Nat. Commun 9:2090 29844387 10.1038/s41467-018-04444-w PMC5974187

[113] Salam AP, Horby P. 2018. Isolation of viable Zika virus from spermatozoa. Lancet Infect. Dis 18:144 10.1016/S1473-3099(18)30020-3 29412962

[114] Paz-Bailey G, Rosenberg ES, Sharp TM. 2019. Persistence of Zika virus in body fluids—final report. N. Engl. J. Med 380:198–99 10.1056/NEJMc1814416 PMC7105955 30625068

[115] Khan S, Woodruff EM, Trapecar M, Fontaine KA, Ezaki A, 2016. Dampened antiviral immunity to intravaginal exposure to RNA viral pathogens allows enhanced viral replication. J. Exp. Med 213:2913–29 27852793 10.1084/jem.20161289 PMC5154948

[116] Dudley DM, Newman CM, Lalli J, Stewart LM, Koenig MR, 2017. Infection via mosquito bite alters Zika virus tissue tropism and replication kinetics in rhesus macaques. Nat. Commun 8:2096 29235456 10.1038/s41467-017-02222-8 PMC5727388

[117] Yockey LJ, Varela L, Rakib T, Khoury-Hanold W, Fink SL, 2016. Vaginal exposure to Zika virus during pregnancy leads to fetal brain infection. Cell 166:1247–56.e4 27565347 10.1016/j.cell.2016.08.004 PMC5006689

[118] Scott JM, Lebratti TJ, Richner JM, Jiang X, Fernandez E, 2018. Cellular and humoral immunity protect against vaginal Zika virus infection in mice. J. Virol 92:e00038–18 29343577 10.1128/JVI.00038-18 PMC5972878

[119] Teixeira FME, Pietrobon AJ, Oliveira LM, Oliveira L, Sato MN. 2020. Maternal-fetal interplay in Zika virus infection and adverse perinatal outcomes. Front. Immunol 11:175 32117303 10.3389/fimmu.2020.00175 PMC7033814

[120] King NJC, Teixeira MM, Mahalingam S. 2017. Zika virus: mechanisms of infection during pregnancy. Trends Microbiol. 25:701–2 28578821 10.1016/j.tim.2017.05.005

[121] Schumacher A, Brachwitz N, Sohr S, Engeland K, Langwisch S, 2009. Human chorionic gonadotropin attracts regulatory T cells into the fetal-maternal interface during early human pregnancy. J. Immunol 182:5488–97 19380797 10.4049/jimmunol.0803177

[122] Lesteberg KE, Fader DS, Beckham JD. 2020. Pregnancy alters innate and adaptive immune responses to Zika virus infection in the reproductive tract. J. Immunol 205:3107–21 33127823 10.4049/jimmunol.2000882 PMC7686295

[123] Jurado KA, Simoni MK, Tang Z, Uraki R, Hwang J, 2016. Zika virus productively infects primary human placenta-specific macrophages. JCI Insight 1:e88461 27595140 10.1172/jci.insight.88461 PMC5007065

[124] Weisblum Y, Oiknine-Djian E, Vorontsov OM, Haimov-Kochman R, Zakay-Rones Z, 2017. Zika virus infects early- and midgestation human maternal decidual tissues, inducing distinct innate tissue responses in the maternal-fetal interface. J. Virol 91:e01905–16 27974560 10.1128/JVI.01905-16 PMC5286880

[125] Simoni MK, Jurado KA, Abrahams VM, Fikrig E, Guller S. 2017. Zika virus infection of Hofbauer cells. Am. J. Reprod. Immunol 77:12613 10.1111/aji.12613 PMC5299062 27966815

[126] Rabelo K, de Souza LJ, Salomao NG, Machado LN, Pereira PG, 2020. Zika induces human placental damage and inflammation. Front. Immunol 11:2146 32983175 10.3389/fimmu.2020.02146 PMC7490298

[127] Bhatnagar J, Rabeneck DB, Martines RB, Reagan-Steiner S, Ermias Y, 2017. Zika virus RNA replication and persistence in brain and placental tissue. Emerg. Infect. Dis 23:405–14 27959260 10.3201/eid2303.161499 PMC5382738

[128] Adams Waldorf KM, Nelson BR, Stencel-Baerenwald JE, Studholme C, Kapur RP, 2018. Congenital Zika virus infection as a silent pathology with loss of neurogenic output in the fetal brain. Nat. Med 24:368–74 29400709 10.1038/nm.4485 PMC5839998

[129] Gurung S, Reuter N, Preno A, Dubaut J, Nadeau H, 2019. Zika virus infection at mid-gestation results in fetal cerebral cortical injury and fetal death in the olive baboon. PLOS Pathog. 15:e1007507 30657788 10.1371/journal.ppat.1007507 PMC6355048

[130] Seferovic M, Sanchez-San Martin C, Tardif SD, Rutherford J, Castro ECC, 2018. Experimental Zika virus infection in the pregnant common marmoset induces spontaneous fetal loss and neurodevelopmental abnormalities. Sci. Rep 8:6851 29717225 10.1038/s41598-018-25205-1 PMC5931554

[131] Bohm EK, Vangorder-Braid JT, Jaeger AS, Moriarty RV, Baczenas JJ, 2021. Zika virus infection of pregnant *Ifnar1*^−/−^ mice triggers strain-specific differences in fetal outcomes. J. Virol 95:e0081821 34379510 10.1128/JVI.00818-21 PMC8513483

[132] Yockey LJ, Jurado KA, Arora N, Millet A, Rakib T, 2018. Type I interferons instigate fetal demise after Zika virus infection. Sci. Immunol 3:1680 10.1126/sciimmunol.aao1680 PMC6049088 29305462

[133] Xu P, Gao J, Shan C, Dunn TJ, Xie X, 2021. Inhibition of innate immune response ameliorates Zika virus-induced neurogenesis deficit in human neural stem cells. PLOS Negl. Trop. Dis 15:e0009183 33657175 10.1371/journal.pntd.0009183 PMC7959377

[134] Valerdi KM, Hage A, van Tol S, Rajsbaum R, Giraldo MI. 2021. The role of the host ubiquitin system in promoting replication of emergent viruses. Viruses 13:369 33652634 10.3390/v13030369 PMC7996891

[135] Byk LA, Iglesias NG, De Maio FA, Gebhard LG, Rossi M, Gamarnik AV. 2016. Dengue virus genome uncoating requires ubiquitination. mBio 7:e00804–16 27353759 10.1128/mBio.00804-16 PMC4937216

[136] Wang S, Liu H, Zu X, Liu Y, Chen L, 2016. The ubiquitin-proteasome system is essential for the productive entry of Japanese encephalitis virus. Virology 498:116–27 27567260 10.1016/j.virol.2016.08.013

[137] Giraldo MI, Xia H, Aguilera-Aguirre L, Hage A, van Tol S, 2020. Envelope protein ubiquitination drives entry and pathogenesis of Zika virus. Nature 585:414–19 32641828 10.1038/s41586-020-2457-8 PMC7501154

[138] Choy MM, Sessions OM, Gubler DJ, Ooi EE. 2015. Production of infectious dengue virus in *Aedes aegypti* is dependent on the ubiquitin proteasome pathway. PLOS Negl. Trop. Dis 9:e0004227 26566123 10.1371/journal.pntd.0004227 PMC4643912

[139] Choy MM, Zhang SL, Costa VV, Tan HC, Horrevorts S, Ooi EE. 2015. Proteasome inhibition suppresses dengue virus egress in antibody dependent infection. PLOS Negl. Trop. Dis 9:e0004058 26565697 10.1371/journal.pntd.0004058 PMC4643959

[140] Giraldo MI, Hage A, van Tol S, Rajsbaum R. 2020. TRIM proteins in host defense and viral pathogenesis. Curr. Clin. Microbiol. Rep 7:101–14 32837832 10.1007/s40588-020-00150-8 PMC7414267

[141] Hage A, Rajsbaum R. 2019. To TRIM or not to TRIM: the balance of host-virus interactions mediated by the ubiquitin system. J. Gen. Virol 100:1641–62 31661051 10.1099/jgv.0.001341 PMC7011758

[142] Lin M, Zhao Z, Yang Z, Meng Q, Tan P, 2016. USP38 inhibits type I interferon signaling by editing TBK1 ubiquitination through NLRP4 signalosome. Mol. Cell 64:267–81 27692986 10.1016/j.molcel.2016.08.029

[143] Zhao Z, Su Z, Liang P, Liu D, Yang S, 2020. USP38 couples histone ubiquitination and methylation via KDM5B to resolve inflammation. Adv. Sci 7:2002680 10.1002/advs.202002680 PMC7675183 33240782

[144] Wang Y, Li Q, Hu D, Gao D, Wang W, 2021. USP38 inhibits Zika virus infection by removing envelope protein ubiquitination. Viruses 13:2029 34696459 10.3390/v13102029 PMC8538320

[145] DiGiacomo V, Meruelo D. 2016. Looking into laminin receptor: critical discussion regarding the non-integrin 37/67-kDa laminin receptor/RPSA protein. Biol. Rev. Camb. Philos. Soc 91:288–310 25630983 10.1111/brv.12170 PMC5249262

[146] Hu D, Wang Y, Li A, Li Q, Wu C, Shereen MA, 2021. LAMR1 restricts Zika virus infection by attenuating the envelope protein ubiquitination. Virulence 12:1795–807 34282707 10.1080/21505594.2021.1948261 PMC8293954

[147] Wang H, Meng H, Li X, Zhu K, Dong K, 2017. PELI1 functions as a dual modulator of necroptosis and apoptosis by regulating ubiquitination of RIPK1 and mRNA levels of c-FLIP. PNAS 114:11944–49 29078411 10.1073/pnas.1715742114 PMC5692605

[148] Luo H, Li G, Wang B, Tian B, Gao J, 2020. Peli1 signaling blockade attenuates congenital zika syndrome. PLOS Pathog. 16:e1008538 32544190 10.1371/journal.ppat.1008538 PMC7297310

[149] Nambala P, Yu WY, Lo YC, Lin CW, Su WC. 2020. Ubiquitination of Zika virus precursor membrane protein promotes the release of viral proteins. Virus Res. 286:198065 32574678 10.1016/j.virusres.2020.198065

[150] van den Boom J, Meyer H. 2018. VCP/p97-mediated unfolding as a principle in protein homeostasis and signaling. Mol. Cell 69:182–94 29153394 10.1016/j.molcel.2017.10.028

[151] Ramanathan HN, Zhang S, Douam F, Mar KB, Chang J, 2020. A sensitive yellow fever virus entry reporter identifies valosin-containing protein (VCP/p97) as an essential host factor for flavivirus uncoating. mBio 11:e00467–20 32291299 10.1128/mBio.00467-20 PMC7157815

[152] Wang L, Moreira EA, Kempf G, Miyake Y, Oliveira Esteves BI, 2022. Disrupting the HDAC6-ubiquitin interaction impairs infection by influenza and Zika virus and cellular stress pathways. Cell Rep. 39(4):110736 35476995 10.1016/j.celrep.2022.110736 PMC9065369

